# Flight-related determinants of health-related quality of life of asylum seekers and refugees in Germany: a longitudinal study based on the German Socio-Economic Panel (SOEP)

**DOI:** 10.1186/s12889-024-19489-4

**Published:** 2024-07-23

**Authors:** Thomas Grochtdreis, Hans-Helmut König, Judith Dams

**Affiliations:** Department of Health Economics and Health Services Research, Hamburg Center for Health Economics, University Medical Center Hamburg-Eppendorf, Martinistr. 52, 20246 Hamburg, Germany

**Keywords:** Health, Quality of life, Psychological well-being, Social determinants of health, Refugees

## Abstract

**Background:**

Germany played a key role as receiving country during the so-called refugee and displacement crisis with about 5 million asylum seekers arriving in the EU between 2014 and 2020. It is well known that asylum seekers and refugees (ASRs) have a high burden of disease and are particularly prone to mental disorders such as trauma, stress-related and affective disorders. Not much is known about the determinants of health-related quality of life (HrQoL) among ASRs, especially in the context of the flight. Therefore, the aim of this study was to analyze the associations between flight-related characteristics and HrQoL of ASRs in Germany.

**Methods:**

The sample of this study was based on five consecutive waves of the Survey of Refugees samples of the German Socio-Economic Panel (*n* = 8015; 14,314 observations). Mental and physical HrQoL was measured using the mental (MCS) and physical (PCS) component summary scores of the SF-12v2. Associations between flight-related characteristics and HrQoL were examined using multilevel mixed-effects linear regressions.

**Results:**

The different countries of birth were associated with varying MCS and PCS scores. The MCS and PCS scores were lower among ASRs with an economic situation below average in their countries of origin. Persecution, discrimination, and poor living conditions as reasons for leaving the county were associated with lower MCS scores. ASRs who were dissatisfied with their own living situation and who were discriminated often due to their origin had both lower MCS and PCS scores. Not feeling welcome in Germany and missing people from one’s country of origin were both associated with lower MCS scores. No worries about not being able to stay in Germany or not being able to return to one’s country of origin were both associated with higher MCS scores.

**Conclusions:**

The economic situation in the country of origin and the presence of persecution, discrimination, and/or poor living conditions as reason for flight may be pre-flight-related determinants of HrQoL of ASRs in Germany. Possible post-flight-related determinants can be the residence status, the satisfaction with one’s living situation, discrimination due to one’s origin and a feeling of missing people from one’s country of origin. With regard to those determinants, the clarity about the residence status, reducing racial discrimination and the mourning of flight-related circumstances must be ensured.

**Supplementary Information:**

The online version contains supplementary material available at 10.1186/s12889-024-19489-4.

## Background

Between 2014 and 2020, about 5 million asylum seekers arrived in the EU [1]. Together with Italy, France and Sweden, Germany played a key role as a receiving country during the so-called refugee and displacement crisis that began around 2015 [2]. During the above-mentioned period, 38% of all asylum seekers in the EU arrived in Germany [1]. For 2020, about 1,850,000 asylum seekers and refugees (ASRs) were registered in the German Central Register of Foreigners (“Ausländerzentralregister”), of whom 75% were entitled to asylum and 25% were (rejected) asylum seekers [3]. An asylum seeker is defined as a person who *“leaves to stay in a foreign country on the grounds of fear of persecution or actual persecution/serious harm in the country of origin*” and especially in the EU, a refugee is defined as “*a person who has specifically sought and received legal asylum*” [4].

Migration is an important determinant in explaining differences in population health [5]. However, any resulting health advantages and disadvantages cannot be attributed to migration alone, but occur together with other social determinants that influence health. According to the International Organization for Migration, migrants who are displaced, fleeing disasters or conflicts, in particular, are placed in situations that may affect their mental and physical health and probably also their health-related quality of life (HrQoL) [6]. Thereby, HrQoL may be defined as “*how well a person functions in their life and his or her perceived well-being in physical, mental, and social domains of health*” [7]. Consequently, HrQoL must be distinguished from health measured based on clinical symptoms, as health is described in terms of functioning and well-being [8, 9].

Furthermore, the pre-flight and movement phases play a role in the ASRs’ health and well-being, e.g. through pre-flight events and trauma, as well as the circumstances and conditions of their flight. In addition, during the arrival and integration phases of the flight, the ASRs’ health and well-being are influenced by social determinants, including social exclusion, discrimination, language, cultural values, and separation from family/partner [6]. It is well known that ASRs in the EU have a high burden of disease and are particularly prone to mental disorders such as trauma, stress-related and affective disorders (e.g. [10–16]). Yet, the burden of disease is relatively heterogeneous among ASRs with regard to the flight-related and sociodemographic characteristics country of origin, residence status, lack of current information about family members left behind, gender, age, marital status, region of residence and socio-economic status [11–15]. Furthermore, the health (care)-related characteristics number of traumatic events, number of mental disorders, subjective need for health care, and need for support in the health care system were associated with the burden of disease among ASRs in the EU [15, 16].

However, only a small number of studies described the HrQoL and quality of life (QoL) of the general population of ASRs in the EU who arrived during the so-called refugee and displacement crisis [17–21]. The WHO defines QoL as “*an individual’s perception of their position in life in the context of the culture and value systems in which they live and in relation to their goals*,* expectations*,* standards and concerns*” [22]. The concept of HrQoL, which is related to the concept of QoL, encompasses those aspects of QoL that include an individual’s perception of their mental and physical state of health [23–25]. Among Syrian ASRs who arrived in Sweden, the QoL based on the WHOQOL-BREF, an abbreviated version of the cross-cultural well-being assessment instrument WHOQOL-100 [26], was rated below the Swedish general population norms in the mental and physical health domains [21, 27]. Among Syrian ASRs who arrived in Germany, separation from marital partners was associated with a lower QoL in the mental health domain of the WHOQOL-BREF [19]. Compared with normative values for the general German population, the mental and physical HrQoL measured by the mental (MCS) and physical (PCS) component summary scores of the Short-Form 12 Item Version 2 (SF-12v2), a short questionnaire derived from the SF-36 to determine HrQoL [28, 29], were lower and higher, respectively [17, 30]. Male gender and employment of ASRs were associated with a higher mental and physical HrQoL, and age, marital status and the country of origin were identified as potential determinants of HrQoL.

Beyond the common determinants of HrQoL, such as the sociodemographic characteristics gender, age, and socio-economic status, not much is known about the associations between flight-related characteristics and HrQoL. Among persons with direct migration background in Germany, one study found associations between the country of origin, age at immigration, and physical HrQoL [31]. Another study identified the time since migration to Germany, the relationship status before migration, connectedness with Germany, disadvantages due to origin, and oral ability in the German language as potential determinants of HrQoL among persons with direct migration background [32]. To our knowledge, only one study has analyzed flight-related determinants of HrQoL among ASRs [18]. This study by Sengoelge et al. [18], which examined the associations between post-migration stressors and generic HrQoL measured by the EQ-5D-5L, the five level-version of the EQ-5D covering the five dimensions of HrQoL: mobility, self-care, usual activities, pain/discomfort, and anxiety/depression [33], of Syrian ASRs who arrived in Sweden, found negative associations between financial strain, social strain and HrQoL. For Germany, however, apart from the findings mentioned above, flight-related determinants of HrQoL among ASRs who arrived in Germany after 2015 are not known. In order to influence the individual perception of the mental and physical health state of ASRs positively, it is necessary to examine more closely, which flight-related characteristics are possible determinants of HrQoL. Thus, the aim of this study was to analyze the associations between pre- and post-flight-related characteristics and HrQoL of ASRs in Germany and to identify possible flight-related determinants of HrQoL.

## Methods

### Sample

The German Socio-Economic Panel (SOEP) is a representative German household panel with about 30,000 participants in almost 15,000 households assessed annually by the German Institute for Economic Research (DIW Berlin) since 1984. Since 2016, a Survey of Refugees has been integrated into the SOEP based on a cooperation between the German Institute for Employment Research, the SOEP at the DIW Berlin, and the Research Centre on Migration, Integration, and Asylum of the German Federal Office for Migration and Refugees [34]. The Survey of Refugees consists of three subsamples (M3, M4, M5) drawn from the German Central Register of Foreigners using a two-stage clustered disproportional stratified sampling design. The M3 and M4 subsamples were introduced in 2016 with 1698 and 1638 households, respectively, and the M5 enlargement sample introduced in 2017 added 1519 households.

By 2022, five consecutive waves of the M3 to M5 subsamples were available (waves 33 to 37). The persons from these five waves were used to generate a longitudinal sample of ASRs (*n* = 18,836; 70,691 observations). The sample of ASRs was restricted to those observations without missing information on HrQoL, age and country of origin (*n* = 8015; 14,314 observations). This exclusion of so many observations is plausible, as the SF-12v2 that has been used for measuring HrQoL is generally only available for the adult sample and the waves of even years in the SOEP and data on the SF-12v2 were only available in exceptional cases for the odd years (2017 and 2019).

### Measures

The HrQoL was measured in the SOEP using a modified version of the SF-12v2 [28, 29]. The SF-12v2 consists of 12 items that can be summarized into the eight subscales ‘physical functioning’, ‘physical role limitations’, ‘bodily pain’, ‘general health’, ‘vitality’, ‘social functioning’, ‘emotional role limitations’ and ‘mental health’ [28, 30]. In contrast to version 1, three to five response categories are specified for all items in the SF-12v2 [35]. In the SOEP, the SF-12v2 was modified by replacing the original question about ‘work interference due to pain’ with a question from the SF-36 about ‘severe physical pain’ [35, 36]. In addition, the SF-12v2 questionnaire was adapted in terms of layout, form, and order of the questions.

The mental and physical HrQoL were represented by the MCS and PCS scores of the SF-12v2. The MCS score was calculated using the Z-transformed subscales ‘social functioning’, ‘emotional role limitations’, and ‘mental health’. The PCS score was calculated using the Z-transformed subscales ‘physical functioning’, ‘physical role limitations’, ‘social functioning’, and ‘bodily pain’. Due to the differences between the original SF12-v2 and the modified version in the SOEP [28, 29, 35], a norm-based scoring using mean values and standard deviations of a German normative sample based on the SOEP was used for Z-transformation instead of the US standard sample [35, 36]. Thus, the MCS and PCS scores represent mental and physical HrQoL on a scale between 0 (worst possible HrQoL) and 100 (best possible HrQoL).

The HrQoL using preference-weighted data was represented by the SF-6D index score. The SF-12v2 subscales ‘physical functioning’, ‘role limitations’, ‘social functioning’, ‘pain’, ‘mental health’, and ‘vitality’, which have three to five ordinal levels, can theoretically define 7500 different health states [37, 38]. Using preference weights derived from the UK general population, index scores were estimated for each possible health state on a scale between 0 (death) and 1 (full health).

Gender, age, religious affiliation, and country of origin were derived from the SOEP as sociodemographic characteristics. The country of origin was categorized based on the geographical regions of the Standard Country or Area Codes for Statistical Use (M49) of the United Nations Statistics Division [39]. The following self-reported pre-flight-related characteristics were derived from the SOEP: economic situation in the country of origin (How would you assess your economic situation at that time compared to the situation of others in your country? (Well) above average, average, (well) below average), connectedness with the country of origin (And how strongly do you feel connected with your country of origin? (Very) strong, in some respects, hardly/not at all). In addition, fear of violent conflict/war, persecution, discrimination, poor living conditions, and the economic situation were asked as reasons for leaving the country of origin (What were the decisive reasons for you to leave this country?), whereby multiple answers were possible. As self-reported post-flight-related characteristics, the time since flight to Germany (When did you move to Germany?), the residence status (What residence permit do you currently have?), satisfaction with living situation (How satisfied were you with your living situation at that time? (Completely) satisfied, neither satisfied nor dissatisfied, (completely) dissatisfied), and discrimination due to origin (How often have you personally experienced being disadvantaged in Germany because of your origin? Often, rarely, never) were derived from the SOEP. It was also asked about one’s feeling of missing people from the country of origin (How often do you feel that you miss people from your country of origin? Very often, sometimes, rarely or never) and the feeling of being welcome (Did you feel welcome when you arrived in Germany? Completely/predominantly, in some respects, barely/never). Furthermore, it was asked about worries about not being able to stay in Germany (Do you worry that you will be unable to stay in Germany? I worry a lot, I worry somewhat, I don’t worry at all) and worries about not being able to return to the country of origin (Are you worried that you will be unable to return to your country of origin? I worry a lot, I worry somewhat, I don’t worry at all).

All interview materials were translated from German into seven languages (English, Arabic, Farsi, Pashto, Urdu, and Kurmanji) that were relevant for the respondents who were expected to have insufficient German language skills [34, 40]. Furthermore, if necessary, the opportunity to take an interpreter to the interviews was given.

### Statistical analysis

Missing information on the 24 variables used varied between 0.00% and 5.57%, and 4060 of all 343,536 records (1.18%) were incomplete. Multiple imputation by chained equations with predictive mean matching and a total number of m = 20 imputations was used to improve accuracy and statistical power of the analyses.

Descriptive statistics of sociodemographic and flight-related characteristics were calculated based on cross-sectional data, using persons’ data at first occurrence in the selected analytical sample. Mean MCS, PCS, and SF-6D index scores were calculated in relation to sociodemographic and flight-related characteristics, and differences in means were analyzed using F-tests.

Associations between the scores of the MCS, PCS, and SF-6D index and flight-related characteristics were examined using multilevel mixed-effects linear regressions. The personal identifier was used as a random effect in the regression and cluster robust standard errors were calculated. The sociodemographic characteristics gender, age, country of origin, and religious affiliation were added as covariates to the regression models. The pre-flight-related characteristics ‘economic situation in the country of origin’, ‘connectedness with country of origin’ and the reasons for leaving the country of origin were added. As post-flight-related characteristics, the time since flight to Germany, the residence status, ‘satisfaction with living situation’, ‘discrimination due to origin’, ‘feeling of being welcome’, ‘feeling of missing people from the country of origin’, ‘worries about not being able to stay in Germany’ and ‘worries about not being able to return to country of origin’ were added to the regression models. Furthermore, the survey year and the subsample were taken into account as covariates.

As the COVID-19 pandemic, which was declared in March 2020 by the WHO, may have had an impact on the HrQoL of ASRs, a sensitivity analysis was conducted by restricting the data to the survey years 2016 to 2019. All analyses were performed using Stata/MP 18.0 (StataCorp, College Station, Texas, USA). All statistics were two-sided with a significance level of *p* < 0.05.

## Results

### Sample characteristics

Sociodemographic characteristics and mean PCS, MCS and SF-6D index scores of the sample (*n* = 8015) are shown in Table 1. The mean age of the sample was 33 years and 51% were female. The majority of the sample was born in Syria (52%). Furthermore, 14% of the sample was born in Iraq and 12% in Afghanistan. The largest part of the sample was affiliated with a Muslim religion (73%).

The mean MCS and PCS score of the total sample was 48.22 and 53.15, respectively. The mean SF-6D index score was 0.73. The mean MCS, PCS, and SF-6D index scores of the subsamples in relation to sociodemographic characteristics and flight-related characteristics are shown in Tables S1 and S2 [see Supplementary Material 1].

**Table 1:** Sociodemographic characteristics of the sample (years 2016 to 2020; *n* = 8015)

Variables	*N* (%) / Mean (SE)
Sex, female	4096 (51.10)
Age, mean	32.95 (0.12)
18–24	2149 (26.81)
25–34	2708 (33.79)
35–44	1895 (23.64)
≥ 45	1263 (15.76)
Country of origin	
Syria	4188 (52.25)
Iraq	1139 (14.21)
Afghanistan	959 (11.97)
Europe^a^	417 (5.20)
Africa^b^	701 (8.75)
Other Asia^c^	611 (7.62)
Religious affiliation	
Muslim	5839 (72.85)
Other religion or non-denominational	2176 (27.15)
MCS	48.22 (0.13)
PCS	53.15 (0.11)
SF-6D index	0.73 (0.00)

SE: Standard error

^a^ Russia, Serbia, Albania, Kosovo, Macedonia, Ukraine, Bosnia/Herzegovina, Romania, Montenegro, Moldova

^b^ Eritrea, Somalia, Nigeria, Ethiopia, Libya, Gambia, Sudan, Egypt, Guinea, Cameroon, Ghana, Morocco, Algeria, Congo, Chad, Ivory Coast, Senegal, Tunisia, Kenya, Burkina Faso, Mali, Niger, Angola, Uganda, Sierra Leone, Rwanda

^c^ Iran, Pakistan, Lebanon, Armenia, Georgia, Saudi Arabia, Kuwait, Azerbaijan, Palestine, Turkey, Sri Lanka, United Arab Emirates, Bangladesh, India, Tajikistan, Jordan, Yemen, Kyrgyzstan, Uzbekistan Mongolia, Nepal, Kazakhstan, Myanmar

### Flight-related characteristics

In Table 2, flight-related characteristics of the sample are shown. The main reason for leaving the country of origin was fear of violent conflict/war (78%), followed by persecution (47%), discrimination (42%), and the economic situation (29%). An average and below average economic situation in the country of origin was reported by 48% and 27% of all persons in the sample, respectively. The majority of the sample had a (very) strong connectedness with their country of origin (48%). The mean time since flight to Germany was about two years.

The residence status of the persons in the sample was mostly accompanied by temporary residence permissions (62%) or without residence permissions (31%). With regard to post-migration stress, the majority of persons in the sample was satisfied with their living situation (56%), has never been discriminated due to their origin (64%), and felt completely or predominantly welcome in Germany (85%). However, about 61% of all persons in the sample had a feeling of missing people from their country of origin. About 40% of all persons in the sample were not worried about not being able to stay in Germany, yet about 38% were very worried.

**Table 2:** Flight-related characteristics of the sample (years 2016 to 2020; *n* = 8015)

Variables	*N* (%) / Mean (SE)
Economic situation in country of origin	
Below average	2153 (26.86)
Average	3837 (47.87)
Above average	2026 (25.27)
Connectedness with country of origin	
(Very) strong	3882 (48.44)
In some respects	2273 (28.36)
Hardly or not at all	1860 (23.20)
Reason for leaving country of origin^a^	
Fear of violent conflict/war, yes	6266 (78.18)
Persecution, yes	3794 (47.33)
Discrimination, yes	3351 (41.81)
Poor living conditions, yes	2981 (37.19)
Economic situation, yes	2325 (29.01)
Time since flight to Germany, years	1.91 (0.02)
Residence status^b^	
No residence permission^c^	2477 (30.90)
Temporary residence permission^d^	4960 (61.88)
Permanent residence permission^e^	220 (2.75)
Satisfaction with living situation	
Dissatisfied	1304 (16.27)
Neither satisfied nor dissatisfied	2201 (27.46)
Satisfied	4510 (56.27)
Discrimination due to origin	
Often	533 (6.65)
Rarely	2330 (29.07)
Never	5152 (64.28)
Feeling of missing people from the country of origin	
(Very) often	4863 (60.67)
Sometimes	1799 (22.44)
Rarely or never	1353 (16.88)
Feeling of being welcome in Germany	
Completely or predominantly	6795 (84.77)
In some respects	902 (11.26)
Barely or not at all	318 (3.97)
Worries about not being able to stay in Germany	
Great worries	3028 (37.78)
Some worries	1773 (22.12)
No worries	3214 (40.10)
Worries about not being able to return to country of origin	
Great worries	1311 (16.36)
Some worries	2134 (26.62)
No worries	4570 (57.02)

SE: Standard error

^a^ Multiple answers possible

^b^ Another/no residence status not shown

^c^ Permission to stay pursuant to Sect. 55 of the German Asylum Law (asylum seekers) or temporary suspension of deportation according to section 60a of the German Residence Act

^d^ Settlement permit according to Sect. 26 sub-Sect. 3 of the German Residence Act

^e^ Residence permit according to Sect. 25 sub-Sect. 1 of the German Residence Act (persons entitled to asylum), according to Sect. 25 sub-Sect. 2 of the German Residence Act (persons with refugee status), according to Sect. 22 or Sect. 23 of the German Residence Act (admission on humanitarian grounds), or residence permit pursuant to § 23a or § 25 sub-Sects. 3, 4 or 5 of the German Residence Act (admission on other humanitarian grounds)

### Regression analysis

The results of the multilevel mixed-effects linear regressions of MCS and PCS scores are shown in Table 3. MCS and PCS scores were not associated with gender and religious affiliation. A higher age was statistically significantly associated with lower MCS (− 0.07) and PCS (− 0.38) scores, both with *p* < 0.001. Being born in Afghanistan (− 1.27, *p* < 0.001) and other Asian countries (− 1.08, *p* = 0.020) was statistically significantly associated with a lower MCS score compared with being born in Syria, whereas being born in an African country was statistically significantly associated with a higher MCS score (+ 2.63; *p* < 0.001). A statistically significantly lower PCS score was associated with being born in Iraq (− 1.19, *p* < 0.001), Afghanistan (− 1.30, *p* < 0.001), and in European countries (− 1.34, *p* = 0.012) compared with being born in Syria, while being born in an African country was statistically significantly associated with a higher PCS score (+ 1.27, *p* < 0.001).

**Table 3:** Multilevel mixed-effects linear regressions of MCS and PCS scores and selected sociodemographic and flight-related characteristics with cluster robust standard errors (years 2016 to 2020; *n* = 8015; 14,314 observations)

Variable	Model 1 (dependent variable MCS)			Model 2 (dependent variable PCS)		
	Coeff.	95% CI	*p*-value	Coeff.	95% CI	*p*-value
Gender (Ref. male)						
Female	0.01	−0.34; 0.35	0.966	−0.10	−0.38; 0.18	0.498
Age, years	**−0.07**	**−0.09; −0.06**	**< 0.001**	**−0.38**	**−0.39; −0.36**	**< 0.001**
Country of origin (Ref. Syria)						
Iraq	0.51	−0.12; 1.12	0.113	**−1.19**	**−1.80; −0.59**	**< 0.001**
Afghanistan	**−1.27**	**−1.98; −0.55**	**< 0.001**	**−1.30**	**−1.94; −0.67**	**< 0.001**
Europe	0.25	−0.93; 1.42	0.680	**−1.34**	**−2.38; −0.30**	**0.012**
Africa	**2.63**	**1.85; 3.40**	**< 0.001**	**1.27**	**0.59; 1.95**	**< 0.001**
Other Asia^a^	**−1.08**	**−1.98; −0.17**	**0.020**	−0.62	−1.41; 0.16	0.117
Religious affiliation (Ref. other religion or non-denominational)						
Muslim	−0.42	−0.92; 0.07	0.094	−0.15	−0.59; 0.29	0.504
Economic situation in country of origin (Ref. average)						
Above average	**0.54**	**0.07; 1.01**	**0.024**	**0.45**	**0.02; 0.89**	**0.041**
Below average	**−0.75**	**−1.25; −0.25**	**0.003**	**−0.65**	**−1.12; −0.19**	**0.006**
Connectedness with country of origin (Ref. in some respects)						
(Very) strong	0.17	−0.26; 0.60	0.450	**0.46**	**0.08; 0.83**	**0.017**
Hardly or not at all	−0.17	−0.69; 0.34	0.510	−0.40	−0.85; 0.05	0.079
Reason for leaving country of origin (Ref. no/not applicable)						
Fear of violent conflict/war	0.12	−0.44; 0.68	0.679	0.21	−0.28; 0.71	0.395
Persecution	**−0.51**	**−0.93; −0.09**	**0.018**	**0.57**	**0.17; 0.96**	**0.005**
Discrimination	**−0.52**	**−0.97; −0.08**	**0.022**	0.24	−0.18; 0.66	0.255
Poor living conditions	**−0.77**	**−1.26; −0.28**	**0.002**	−0.40	−0.85; 0.05	0.085
Economic situation	−0.18	−0.70; 0.33	0.485	−0.03	−0.52; 0.46	0.897
Time since flight to Germany, years	−0.10	−0.92; 0.07	0.094	0.10	−0.04; 0.24	0.174
Residence status^b^ (Ref. no residence permission^c^)						
Temporary residence permission^d^	**1.24**	**0.69; 1.79**	**< 0.001**	**−1.07**	**−1.52; −0.61**	**< 0.001**
Permanent residence permission^e^	−0.38	−1.60; 0.84	0.539	−0.85	−1.86; 0.16	0.098
Satisfaction with living situation (Ref. neither satisfied nor dissatisfied)						
Satisfied	**2.22**	**1.80; 2.63**	**< 0.001**	**0.47**	**0.12; 0.82**	**0.008**
Dissatisfied	**−1.70**	**−2.31; −1.09**	**< 0.001**	**−0.63**	**−1.14; −0.13**	**0.014**
Discrimination due to origin (Ref. rarely)						
Never	**1.74**	**1.80; 2.63**	**< 0.001**	**0.55**	**0.20; 0.91**	**0.002**
Often	**−1.70**	**−2.31; −1.09**	**< 0.001**	−0.49	−1.21; 0.22	0.173
Feeling of being welcome (Ref. in some respects)						
Completely or predominantly	**2.07**	**1.44; 2.70**	**< 0.001**	0.38	−0.19; 0.94	0.191
Barely or never	**−2.26**	**−3.52; −1.00**	**< 0.001**	−0.07	−1.19; 1.06	0.908
Feeling of missing people from the country of origin (Ref. sometimes)						
Rarely or never	0.06	−0.57; 0.70	0.844	**0.58**	**0.01; 1.14**	**0.045**
(Very) often	**−1.02**	**−1.50; −0.55**	**< 0.001**	**−0.47**	**−0.92; −0.03**	**0.037**
Worries about not being able to stay in Germany (Ref. some worries)						
Great worries	**−0.67**	**−1.16; −0.17**	**0.009**	−0.16	−0.57; 0.24,	0.431
No worries	**1.71**	**1.26; 2.15**	**< 0.001**	0.37	−0.01; 0.75	0.054
Worries about not being able to return to country of origin (Ref. some worries)						
Great worries	**−1.12**	**−1.73; −0.52**	**< 0.001**	0.04	−0.45; 0.52	0.878
No worries	**1.33**	**0.90; 1.76**	**< 0.001**	**0.57**	**0.22; 0.92**	**0.001**
Survey year (Ref. 2016)						
2017	**0.68**	**0.05; 1.32**	**0.035**	0.04	−0.48; 0.55	0.884
2018	**1.38**	**0.82; 1.93**	**< 0.001**	−0.29	−0.76; 0.18	0.231
2019	**1.96**	**0.65; 3.26**	**0.003**	**−1.17**	**−2.29; −0.05**	**0.040**
2020	**2.81**	**2.07; 3.54**	**< 0.001**	−0.19	−0.88; 0.49	0.577
Subsample	✓			✓		
Constant	**46.17**	**44.79; 47.55**	**< 0.001**	**64.96**	**63.76; 66.15**	**< 0.001**

CI: confidence interval

^a^ Without Syria, Iraq and Afghanistan

^b^ Another/no residence status not shown

^c^ Permission to stay pursuant to Sect. 55 of the German Asylum Law (asylum seekers) or temporary suspension of deportation according to section 60a of the German Residence Act

^d^ Settlement permit according to Sect. 26 sub-Sect. 3 of the German Residence Act

^e^ Residence permit according to Sect. 25 sub-Sect. 1 of the German Residence Act (persons entitled to asylum), according to Sect. 25 sub-Sect. 2 of the German Residence Act (persons with refugee status), according to Sect. 22 or Sect. 23 of the German Residence Act (admission on humanitarian grounds), or residence permit pursuant to § 23a or § 25 sub-Sects. 3, 4 or 5 of the German Residence Act (admission on other humanitarian grounds)

An economic situation in the country of origin above average was statistically significantly associated with both a higher MCS (+ 0.54, *p* = 0.024) and PCS score (+ 0.45, *p* = 0.041), and an economic situation below average was statistically significantly associated with both a lower MCS (− 0.75, *p* = 0.003) and PCS score (− 0.65, *p* = 0.006) compared with an average economic situation. Persecution (− 0.51, *p* = 0.018), discrimination (− 0.52, *p* = 0.022), and poor living conditions (− 0.77, *p* = 0.002) as reasons for leaving the country of origin were statistically significantly associated with lower MCS scores.

The possession of a temporary residence permission was statistically significantly associated with a higher MCS (+ 1.24, *p* < 0.001) and a lower PCS score (− 1.07, *p* < 0.001) compared with no residence permission. Being satisfied with one’s own living situation was statistically significantly associated with both a higher MCS (+ 2.22, *p* < 0.001) and a higher PCS score (+ 0.47, *p* = 0.007), and being dissatisfied was statistically significantly associated with both a lower MCS (− 1.70, *p* < 0.001) and a lower PCS score (− 0.63, *p* = 0.014) compared with being neither satisfied nor dissatisfied.

A frequent discrimination due to one’s origin was statistically significantly associated with a lower MCS score (− 1.70, *p* < 0.001) compared with a rare discrimination. A feeling of not at all being welcome in Germany was statistically significantly associated with a lower MCS score (− 2.26, *p* < 0.001) compared with a feeling of being welcome in some respects. The feeling of missing people from their country of origin (very) often was statistically significantly associated with both a lower MCS (− 1.02) and PCS score (− 0.47, both with *p* ≤ 0.001) compared with a feeling of missing people sometimes. Both having no worries about not being able to stay in Germany and no worries about not being able to return to one’s country of origin were statistically significantly associated with higher MCS scores (+ 1.71 and + 1.33, both with *p* < 0.001) than having some worries.

The results of the multilevel mixed-effects linear regressions of SF-6D index scores are shown in Table S3 [see Supplementary Material 1]. An economic situation in the country of origin above average was statistically significantly associated with a higher SF-6D index score (+ 0.01, *p* = 0.006) and an economic situation below average was statistically significantly associated with a lower SF-6D index score (− 0.01, *p* = 0.002) compared with an average economic situation. Poor living conditions as reason for leaving the country of origin was statistically significantly associated with a lower SF-6D index score (− 0.01, *p* = 0.006). The possession of a permanent residence permission was statistically significantly associated with a lower SF-6D index score (− 0.02, *p* = 0.025) compared with no residence permission. Being satisfied with one’s own living situation was statistically significantly associated with a higher SF-6D index score (+ 0.03, *p* < 0.001), and being dissatisfied was statistically significantly associated with a lower SF-6D index score (− 0.01, *p* = 0.001) compared with being neither satisfied nor dissatisfied. A frequent discrimination due to one’s origin was statistically significantly associated with a lower SF-6D index score (− 0.02, *p* < 0.001) compared with a rare discrimination. A feeling of not at all being welcome in Germany was statistically significantly associated with a lower SF-6D index score (− 0.02, *p* < 0.012) compared with a feeling of being welcome in some respects. The feeling of missing people from their country of origin (very) often was statistically significantly associated with a lower SF-6D index score (− 0.02, *p* < 0.001) compared with a feeling of missing people sometimes. Both having no worries about not being able to stay in Germany and no worries about not being able to return to one’s country of origin were statistically significantly associated with higher SF-6D index score (+ 0.03 and + 0.02, both with *p* < 0.001) than having some worries.

The sensitivity analysis without the COVID-19 pandemic year 2020 did not alter the results of the multilevel mixed-effects linear regression of the MCS and PCS scores (Table S4 [see Supplementary Material 1]).

## Discussion

This was the first study to analyze the associations between HrQoL and pre- and post-flight-related characteristics of ASRs in Germany. It has been shown that the economic situation in the country of origin before the flight determines both the mental and physical HrQoL of ASRs. Furthermore, the presence of persecution, discrimination, and/or poor living conditions as reasons for leaving the country were negatively associated with mental HrQoL. According to a life course model of migration in general and health, the reasons for leaving one’s country of origin can be substantial pre-migration stressors that affect mental and physical health of migrants even later in life after migration [5]. Also, high exposure to potentially traumatic experiences before or during the flight was shown to be associated with lower HrQoL among Syrian ASRs that arrived in Sweden [18].

It was shown in the current study that being born in Afghanistan and other Asian countries was negatively associated with mental HrQoL compared with being born in Syria. Being born in Iraq, Afghanistan, and in European countries was negatively associated with physical HrQoL, and being born in an African country was positively associated with both mental and physical HrQoL. A lower HrQoL of ASRs from Afghanistan and a higher HrQoL of ASRs from Eritrea compared with ASRs from Syria has already been shown elsewhere [17]. Possible explanations for those differences in HrQoL of ASRs from different countries may be different reasons for leaving the country, experiences during the flight as well as post-flight stressors. However, it remains to be seen whether these are real differences in HrQoL or whether differential item functioning may be present and the SF-12v2 performed differently among persons with different origins [41, 42]. Furthermore, social desirability bias might be a reason for differences in HrQoL between ASRs from different countries. It has been shown that ASRs have more desirable response tendencies than persons from the receiving country with regard to their psychopathology [43]. A potential effect of country-specific responses on the perceived pressure to adjust to the local culture and thus answering the SF-12v2 questions in a socially desirable way remains to be investigated.

The possession of a temporary residence permission was positively associated with mental HrQoL compared to not possessing a residence permission. It is known that post-flight stressors, such as delays in processing the asylum application, are important determinants for the development of mental disorders [44–46]. In a systematic review, the possession of a temporary residence permission was negatively associated with mental health compared with a permanent residence permission [47–49]. Furthermore, the residence status was associated with mental health, especially with regard to symptoms of depression and anxiety as well as post-traumatic stress disorder, among ASRs that arrived in Sweden and Switzerland [21, 50]. Unexpectedly, the possession of a temporary residence permission was negatively associated with physical HrQoL compared to not possessing a residence permission. In fact, a more insecure residence permission is related to worse access to health care through language and cultural barriers as well as restricted entitlements [51]. Furthermore, the possession of a temporary residence permission was positively associated with physical QoL among ASRs from Darfur that arrived in Israel [52, 53]. One possible explanation for the result of the current study is that the category of persons not possessing a residence permission contains both persons with a permission to stay pursuant to Sect. 55 of the German Asylum Law (asylum seekers) and persons with a temporary suspension of deportation according to section 60a of the German Residence Act.

Satisfaction with one’s living situation was associated with both the mental and physical HrQoL of ASRs. In the general population, a lower socio-economic status and poorer living conditions were negatively associated with mental health [54, 55]. Post-flight living problems, such as prolonged stays in the refugee housing facilities and poor socio-economic living conditions offered to ASRs, are known to be associated with QoL, disability, physical and mental health throughout Europe, partially even regardless of the residence status [21, 50, 56, 57]. Furthermore, in the current study, a feeling of missing people from one’s country of origin determined both the mental and physical HrQoL, and worries about not being able to stay in Germany and worries about not being able to return to one’s country of origin determined the mental HrQoL of ASRs. It is known that separation from family is associated with negative mental health among ASRs [47, 58, 59]. Furthermore, fewer social contacts, as another indicator of loneliness, were associated with lower mental and physical HrQoL in tortured male ASRs that arrived in Denmark [60, 61].

Discrimination due to one’s origin determined mental and physical HrQoL, and a feeling of being welcome determined the mental HrQoL of ASRs. Among Syrian ASRs that arrived in Sweden, high levels of perceived discrimination, i.e., experiences of unfair treatment based on prejudice, were associated with a lower HrQoL [18]. Experiences of racial discrimination, such as not being welcome in the receiving country, were considered to be a health-threatening stressor for members of stigmatized groups of people who experience disadvantages due to their group membership [62, 63]. According to systematic reviews, racism, and thus racial discrimination, was negatively associated with mental and physical health and QoL [47, 64]. In particular, social exclusion resulting from racial discrimination, was negatively associated with QoL, and perceived racial discrimination was associated with poorer general health and depressive symptoms among ASRs [65–68]. Among migrants in general in Germany, racial discrimination was not only associated with a lower mental and physical HrQoL, but also indicated a causal relationship [69, 70].

### Comparability and generalizability

Compared with the mental (51.8 and 50.9) and physical HrQoL (51.6 and 51.5) measured using the MCS and PCS scores of two other samples of persons with a direct migration background in Germany, the mental (48.2) and physical HrQoL (53.2) of the current sample of ASRs was lower and higher, respectively [32, 71]. The mean age of the ASRs in the current sample was 33 years. This is slightly higher than the mean age of ASRs in Germany who arrived in Germany until 2020 (30 years) according to the German Central Register of Foreigners [3]. The younger age of the ASRs according to the German Central Register of Foreigners can be explained by the fact that 28% of them were minors, whereas the sample of the current study was adults only. Furthermore, according to the German Central Register of Foreigners, only 38.7% of the ASRs who arrived in Germany until 2020 were female, while 51.1% of the ASRs in the current sample were female. Yet, in the Survey of Refugees, a disproportionately high number of women, people over 30, and ASR with a granted residence permit were included in the sample in order to be able to include these relatively small population groups in the study with a sufficient numbers of cases [72]. The Survey of Refugees was designed to be representative of the population of ASRs who arrived in Germany between 2013 and 2016 and were registered in the Central Register of Foreigners [34]. Thus, the generalizability of the current’s study results with regard to the association between flight-related characteristics and HrQoL is possible with the aforementioned oversampling in terms of gender, age, and residence permit.

### Policy implications

Social policies and health care need to take into account all three phases of the life course of ASR, the post-flight phase and, in particular, the impact of the pre-flight and movement phases, as exposures might affect ASRs’ health and HrQoL both acutely and later in life [5]. Thereby, one focus should be the reduction of barriers with regard to access to health care, such as language barriers, cultural stereotypes, or restrictions on insurance coverage and bureaucratic hurdles [32, 73–75].

Particularly in view of the finding that worries about not being able to stay in Germany and the residence status determined HrQoL as well as the burden of disease with regard to mental disorders [76], it is important to ensure clarity regarding the residence status, the prospect of staying and, if necessary, the assurance of a humane repatriation procedure.

In addition, it is crucial not only for the HrQoL of ASRs, but also for society as a whole, that social integration activities are expanded and put on a sound financial footing. Regarding social integration, i.e. the frequency of interactions between individuals from different groups [77, 78], key measures could be to allay concerns about immigration and xenophobia in the German population and to establish social relationships, e.g. by strengthening inter-ethnic social networks and by further consolidating civil society tandem projects, integration caseworkers and social workers [79, 80].

### Strengths and limitations

One major strength of this study was the large sample of ASRs made available by the Survey of Refugees, which was integrated in the SOEP. The sampling of ASR who arrived in Germany for the Survey of Refugees became straightforward, as the DIW Berlin had access to the German Central Register of Foreigners [34]. All interview materials were translated into those languages that were relevant for the respondents who were expected to have insufficient German language skills [34, 40]. Furthermore, the integration of additional flight-related questions with regard to origin, reasons for leaving the country, residence status, and accommodation made it possible to include flight-related characteristics systematically as covariates in the regression models examining associations with mental and physical HrQoL of ASRs in Germany [81].

However, some limitations must also be mentioned. Firstly, the analyses of the current study must be regarded as explanatory rather than confirmatory, as it cannot be proven by an a priori registered research plan that the analytical steps were undertaken without prior knowledge of the results [82]. Due to multiple comparisons, the Type I error may be inflated, so that statistical inference tests must be interpreted conservatively. Secondly, since the flight-related characteristics were largely constant over time, only associations could be analyzed and no conclusions drawn about the causal relationship, despite the availability of data from observations of five waves of the SOEP. Thirdly, an excessive amount of information on education (35%) and on negative experiences during the flight (47%) was missing from the sample of ASRs in the SOEP. Therefore, the important socio-demographic characteristic education and flight-related characteristics that are related to the movement phase were not included in the analyses in order to avoid introducing further uncertainty. Furthermore, no information was available in the SOEP with regard to the actual living situation of the ASRs. Fourthly, as the ASRs provided self-reported information about the flight-related characteristics in the SOEP, social-desirability bias may have been present, which could interfere with the interpretation of the associations with HrQoL. Fifthly, the data of the fifth wave of the SOEP used for the analyses in this study were collected in the first year of the COVID-19 pandemic. However, a sensitivity analysis excluding the fifth wave of the SOEP revealed no evidence of an indirect moderating effect on the associations between flight-related characteristics and HrQoL.

## Conclusion

Pre-flight-related characteristics such as the economic situation and the presence of persecution, discrimination, and/or poor living conditions in the country of origin were found to be determinants of the HrQoL of ASRs after arriving in Germany. After the flight, the residence status and the satisfaction with one’s living situation can be also considered as determinants of HrQoL, as well as discrimination due to one’s origin and a feeling of missing people from one’s country of origin. Clarity about the residence status and social integration must be ensured not only in this respect but also with regard to the association between the prospects of staying in Germany and HrQoL. Further research is needed to investigate the associations between those flight-related characteristics that are related to the movement phase and HrQoL, the causal relationships between flight-related determinants and HrQoL and to analyze the associations between flight-related characteristics and other indicators relevant to health care, such as its service utilization, among ASRs.

### Electronic supplementary material

Below is the link to the electronic supplementary material.

Supplementary Material 1

## Data Availability

The dataset supporting the conclusions of this article can be applied for via the website of the German Institute for Economic Research (DIW), Berlin. It is available free of charge for scientific, non-commercial use. The code used during the current study is available from the corre-sponding author on reasonable request for all interested researchers.
