# Supplementary material for: Flight-related determinants of health-related quality of life of asylum seekers and refugees in Germany: a longitudinal study based on the German Socio-Economic Panel (SOEP)
Source: BMC Public Health. 2024 Jul 23;24:1965. doi: 10.1186/s12889-024-19489-4 (PMC11264381; doi:10.1186/s12889-024-19489-4)
Supplement: Supplementary file 1 — Supplementary Material 1 [file 12889_2024_19489_MOESM1_ESM.docx]

Table S1: Mean MCS, PCS and SF-6D index scores by sociodemographic characteristics (years 2016 to 2020; n = 8015)

|  | **N** | **MCS** | **PCS** | **SF-6D index** |
| --- | --- | --- | --- | --- |
|  |  | Mean (SE) | | |
| Gender |  |  |  |  |
| Female | 4096 | 48.00 (0.18) | 53.16 (0.16) | 0.735 (0.002) |
| Male | 3919 | 48.45 (0.19) | 53.15 (0.16) | 0.731 (0.002) |
| Age |  |  |  |  |
| 18-24 | 2149 | 49.23 (0.25)*** | 57.17 (0.20)*** | 0.773 (0.003)*** |
| 25-34 | 2708 | 48.28 (0.22) | 54.67 (0.18) | 0.746 (0.003) |
| 35-44 | 1895 | 47.95 (0.27) | 51.46 (0.22) | 0.717 (0.003) |
| ≥ 45 | 1263 | 46.80 (0.33) | 45.63 (0.27) | 0.663 (0.004) |
| Country of birth |  |  |  |  |
| Syria | 4188 | 48.68 (0.18)*** | 53.27 (0.16)*** | 0.735 (0.002)*** |
| Iraq | 1139 | 49.12 (0.34) | 52.46 (0.30) | 0.740 (0.005) |
| Afghanistan | 959 | 45.87 (0.37) | 52.34 (0.33) | 0.706 (0.005) |
| Europe | 417 | 46.49 (0.57) | 51.53 (0.50) | 0.709 (0.007) |
| Africa | 701 | 50.26 (0.44) | 55.93 (0.38) | 0.780 (0.006) |
| Other Asia^a^ | 611 | 45.93 (0.47) | 52.85 (0.41) | 0.712 (0.006) |
| Religious affiliation |  |  |  |  |
| Muslim | 5839 | 48.07 (0.15)** | 53.07 (0.13) | 0.744 (0.003)*** |
| Other religion or non-denominational | 2176 | 48.80 (0.25) | 53.39 (0.22) | 0.728 (0.002) |

Comparisons of mean MCS, PCS, and SF-6D index scores by sociodemographic characteristics were analyzed using F tests

MCS: Mental Component Summary; PCS: Physical Component Summary; SE: standard error

^a^ Without Syria, Iraq and Afghanistan

* *p* < 0.05, ** *p* ≤ 0.01, *** *p* ≤ 0.001

Table S2: Mean MCS, PCS and SF-6D index scores by flight-related characteristics (years 2016 to 2020; n = 8015)

|  | **N** | **MCS** | **PCS** | **SF-6D index** |
| --- | --- | --- | --- | --- |
|  |  | Mean (SE) | | |
| Economic situation in country of origin |  |  |  |  |
| Below average | 2153 | 47.10 (0.27)*** | 52.73 (0.23)* | 0.723 (0.004)** |
| Average | 3837 | 48.62 (0.19) | 53.42 (0.17) | 0.739 (0.003) |
| Above average | 2026 | 48.56 (0.25) | 53.08 (0.22) | 0.733 (0.003) |
| Connectedness with country of birth |  |  |  |  |
| (Very) strong | 3882 | 48.00 (0.19) | 53.17 (0.16) | 0.731 (0.002) |
| In some respects | 2273 | 48.35 (0.25) | 53.34 (0.22) | 0.732 (0.003) |
| Hardly or not at all | 1860 | 48.53 (0.27) | 52.90 (0.24) | 0.740 (0.004) |
| Residence title^a^ |  |  |  |  |
| No residence permission^b^ | 2477 | 46.20 (0.23) | 53.54 (0.21) | 0.719 (0.003) |
| Temporary residence permission^c^ | 4960 | 49.31 (0.16) | 53.13 (0.15) | 0.742 (0.002) |
| Permanent residence permission^d^ | 220 | 47.43 (0.79)*** | 51.77 (0.69)** | 0.716 (0.010)*** |
| Satisfaction with living situation |  |  |  |  |
| Dissatisfied | 1304 | 44.71 (0.32)*** | 52.63 (0.28) | 0.699 (0.004)*** |
| Neither satisfied nor dissatisfied | 2201 | 46.96 (0.25) | 53.05 (0.22) | 0.716 (0.003) |
| Satisfied | 4510 | 49.88 (0.17) | 53.36 (0.15) | 0.752 (0.002) |
| Discrimination due to origin |  |  |  |  |
| Often | 533 | 44.38 (0.51)*** | 52.66 (0.45) | 0.692 (0.007)*** |
| Rarely | 2330 | 46.63 (0.24) | 53.39 (0.21) | 0.713 (0.003) |
| Never | 5152 | 49.34 (0.16) | 53.10 (0.14) | 0.747 (0.002) |
| Feeling of missing people from the country of origin |  |  |  |  |
| (Very) often | 4863 | 47.53 (0.17)*** | 52.91 (0.15)* | 0.723 (0.002)*** |
| Sometimes | 1799 | 49.15 (0.28) | 53.37 (0.24) | 0.745 (0.004) |
| Rarely or never | 1353 | 49.46 (0.32) | 53.75 (0.28) | 0.755 (0.004) |
| Feeling of being welcome |  |  |  |  |
| Completely or predominantly | 6795 | 48.98 (0.14)*** | 53.20 (0.12) | 0.742 (0.002)*** |
| In some respects | 902 | 44.77 (0.39) | 53.01 (0.35) | 0.688 (0.005) |
| Barely or not at all | 318 | 41.76 (0.66) | 52.57 (0.58) | 0.665 (0.009) |
| Worries about not being able to stay in Germany |  |  |  |  |
| Great worries | 3028 | 45.94 (0.21)*** | 53.25 (0.19) | 0.716 (0.003)*** |
| Some worries | 1773 | 47.95 (0.28) | 52.80 (0.25) | 0.721 (0.004) |
| No worries | 3214 | 50.52 (0.20) | 53.26 (0.18) | 0.757 (0.003) |
| Worries about not being able to return to country of origin |  |  |  |  |
| Great worries | 1311 | 45.80 (0.32)*** | 52.89 (0.29) | 0.706 (0.004)*** |
| Some worries | 2134 | 47.60 (0.25) | 53.04 (0.22) | 0.720 (0.003) |
| No worries | 4570 | 49.21 (0.17) | 53.28 (0.15) | 0.747 (0.002) |

Comparisons of mean MCS, PCS, and SF-6D index scores by migration-related characteristics were analyzed using F tests

MCS: Mental Component Summary; PCS: Physical Component Summary; SE: standard error

^a^ Another/no residence status not shown

^b^ Permission to stay pursuant to Section 55 of the German Asylum Law (asylum seekers) or temporary suspension of deportation according to section 60a of the German Residence Act

^c^ Settlement permit according to Section 26 sub-section 3 of the German Residence Act

^d^ Residence permit according to Section 25 sub-section 1 of the German Residence Act (persons entitled to asylum), according to Section 25 sub-section 2 of the German Residence Act (persons with refugee status), according to Section 22 or Section 23 of the German Residence Act (admission on humanitarian grounds), or residence permit pursuant to § 23a or § 25 sub-section 3, 4 or 5 of the German Residence Act (admission on other humanitarian grounds)

* *p* < 0.05, ** *p* ≤ 0.01, *** *p* ≤ 0.001

Table S3: Multilevel mixed-effects linear regression of the SF-6D index and selected sociodemographic and flight-related characteristics with cluster robust standard errors (years 2016 to 2020; n = 8015; 14,314 observations)

| Variable | Model 3 (dependent variable SF-6D index) | | |
| --- | --- | --- | --- |
|  | Coeff. | 95% CI | *p*-value |
| Gender (Ref. male) |  |  |  |
| Female | −0.000 | −0.005; 0.004 | 0.915 |
| Age, years | **−0.004** | **−0.004; −0.003** | **< 0.001** |
| Country of origin (Ref. Syria) |  |  |  |
| Iraq | −0.002 | −0.011; 0.006 | 0.586 |
| Afghanistan | **−0.020** | **−0.030; −0.011** | **< 0.001** |
| Europe | −0.003 | −0.019; 0.012 | 0.682 |
| Africa | **0.041** | **0.030; 0.051** | **< 0.001** |
| Other Asia^a^ | **−0.013** | **−0.025; −0.001** | **0.039** |
| Religious affiliation (Ref. other religion or non-denominational) |  |  |  |
| Muslim | **−0.008** | **−0.014; −0.001** | **0.028** |
| Economic situation in country of origin (Ref. average) |  |  |  |
| Above average | **0.009** | **0.003; 0.016** | **0.006** |
| Below average | **−0.011** | **−0.018; −0.004** | **0.002** |
| Connectedness with country of birth (Ref. in some respects) |  |  |  |
| (Very) strong | **0.007** | **0.001; 0.013** | **0.018** |
| Hardly or not at all | −0.003 | −0.010; 0.004 | 0.467 |
| Reason for leaving country of origin |  |  |  |
| Fear of violent conflict/war | 0.002 | −0.005; 0.010 | 0.565 |
| Persecution | 0.001 | −0.005; 0.007 | 0.726 |
| Discrimination | −0.003 | −0.010; 0.003 | 0.270 |
| Living conditions | **−0.010** | **−0.016; −0.003** | **0.006** |
| Economic situation | −0.000 | −0.008; 0.007 | 0.926 |
| Time since flight to Germany, years |  |  |  |
| Residence title^b^ (Ref. no residence permission^c^) |  |  |  |
| Temporary residence permission^d^ | 0.001 | −0.006; 0.009 | 0.719 |
| Permanent residence permission^e^ | **−0.018** | **−0.033; −0.002** | **0.025** |
| Satisfaction with living situation (Ref. neither satisfied nor dissatisfied) |  |  |  |
| Satisfied | **0.029** | **0.024; 0.035** | **< 0.001** |
| Dissatisfied | **−0.013** | **−0.021; −0.006** | **0.001** |
| Discrimination due to origin (Ref. rarely) |  |  |  |
| Never | 0.005 | −0.003; 0.014 | 0.234 |
| Often | **−0.016** | **−0.023; −0.010** | **< 0.001** |
| Feeling of being welcome (Ref. in some respects) |  |  |  |
| Completely or predominantly | **0.030** | **0.021; 0.038** | **< 0.001** |
| Barely or not at all | **−0.019** | **−0.034; −0.004** | **0.012** |
| Feeling of missing people from the country of origin (Ref. sometimes) |  |  |  |
| Rarely or never | 0.005 | −0.003; 0.014 | 0.234 |
| (Very) often | **−0.016** | **−0.023; −0.010** | **< 0.001** |
| Worries about not being able to stay in Germany (Ref. some worries) |  |  |  |
| Great worries | −0.003 | −0.009; 0.004 | 0.389 |
| No worries | **0.026** | **0.020; 0.032** | **< 0.001** |
| Worries about not being able to return to country of origin (Ref. some worries) |  |  |  |
| Great worries | −0.008 | −0.009; 0.004 | 0.389 |
| No worries | **0.022** | **0.016; 0.028** | **< 0.001** |
| Survey year (Ref. 2016) |  |  |  |
| 2017 | **0.010** | **0.002; 0.018** | **0.019** |
| 2018 | **0.010** | **0.003; 0.017** | **0.008** |
| 2019 | 0.011 | −0.006; 0.027 | 0.204 |
| 2020 | **0.018** | **0.007; 0.028** | **0.001** |
| Initial sample | **🗸** | | |
| Constant | **0.782** | **0.763; 0.800** | **< 0.001** |

CI: confidence interval

^a^ Without Syria, Iraq and Afghanistan

^b^ Another/no residence title not shown

^c^ Permission to stay pursuant to Section 55 of the German Asylum Law (asylum seekers) or temporary suspension of deportation according to section 60a of the German Residence Act

^d^ Settlement permit according to Section 26 sub-section 3 of the German Residence Act

^e^ Residence permit according to Section 25 sub-section 1 of the German Residence Act (persons entitled to asylum), according to Section 25 sub-section 2 of the German Residence Act (persons with refugee status), according to Section 22 or Section 23 of the German Residence Act (admission on humanitarian grounds), or residence permit pursuant to § 23a or § 25 sub-section 3, 4 or 5 of the German Residence Act (admission on other humanitarian grounds)

Table S4: Multilevel mixed-effects linear regressions of MCS and PCS scores and selected sociodemographic and flight-related characteristics with cluster robust standard errors (years 2016 to 2019; n = 7765; 11,132 observations): Sensitivity analysis without the first year of the COVID−19 pandemic

| Variable | Model 1 (dependent variable MCS) | | | Model 2 (dependent variable PCS) | | |
| --- | --- | --- | --- | --- | --- | --- |
|  | Coeff. | 95% CI | *p*-value | Coeff. | 95% CI | *p*-value |
| Gender (Ref. male) |  |  |  |  |  |  |
| Female | −0.15 | −0.55; 0.25 | 0.466 | 0.03 | −0.30; 0.36 | 0.8701 |
| Age, years | **−0.09** | **−0.11; −0.07** | **< 0.001** | **−0.37** | **−0.39; −0.35** | **< 0.001** |
| Country of origin (Ref. Syria) |  |  |  |  |  |  |
| Iraq | 0.41 | −0.31; 1.13 | 0.260 | **−1.26** | **−1.91; −0.60** | **< 0.001** |
| Afghanistan | **−1.47** | **−2.27; −0.67** | **< 0.001** | **−1.43** | **−2.12; −0.75** | **< 0.001** |
| Europe | 0.49 | −0.78; 1.75 | 0.451 | **−1.30** | **−2.39; −0.21** | **0.019** |
| Africa | **2.73** | **1.87; 3.59** | **< 0.001** | **1.23** | **0.50; 1.95** | **0.001** |
| Other Asia^a^ | **−1.20** | **−2.18; −0.22** | **0.016** | −0.59 | −1.42; 0.24 | 0.161 |
| Religious affiliation (Ref. other religion or non-denominational) |  |  |  |  |  |  |
| Muslim | −0.41 | −0.96; 0.14 | 0.143 | −0.08 | −0.55; 0.40 | 0.757 |
| Economic situation in country of origin (Ref. average) |  |  |  |  |  |  |
| Above average | 0.44 | −0.08; 0.96 | 0.101 | **0.51** | **0.04; 0.97** | **0.035** |
| Below average | **−1.01** | **−1.59; −0.43** | **0.001** | **−0.65** | **−1.15; −0.16** | **0.010** |
| Connectedness with country of birth (Ref. in some respects) |  |  |  |  |  |  |
| (Very) strong | 0.32 | −0.16; 0.81 | 0.195 | **0.54** | **0.14; 0.95** | **0.008** |
| Hardly or not at all | −0.12 | −0.71; 0.47 | 0.681 | **−0.60** | **−1.08; −0.12** | **0.015** |
| Reason for leaving country of origin |  |  |  |  |  |  |
| Fear of violent conflict/war | 0.18 | −0.44; 0.79 | 0.566 | 0.38 | −0.15; 0.90 | 0.159 |
| Persecution | **−0.55** | **−1.03; −0.07** | **0.025** | **0.58** | **0.15; 1.01** | **0.008** |
| Discrimination | **−0.64** | **−1.14; −0.14** | **0.012** | 0.14 | −0.31; 0.59 | 0.533 |
| Living conditions | **−1.00** | **−1.55; −0.45** | **< 0.001** | −0.39 | −0.88; 0.11 | 0.125 |
| Economic situation | −0.02 | −0.60; 0.56 | 0.943 | −0.04 | −0.57; 0.48 | 0.868 |
| Time since flight to Germany, years | −0.14 | −0.31; 0.02 | 0.092 | **0.08** | −0.07; 0.23 | **0.311** |
| Residence title^b^ (Ref. no residence permission^c^) |  |  |  |  |  |  |
| Temporary residence permission^d^ | **1.15** | **0.56; 1.75** | **< 0.001** | −1.12 | −1.60; −0.66 | **< 0.001** |
| Permanent residence permission^e^ | 0.16 | −1.36; 1.68 | 0.833 | −1.27 | −2.58; 0.04 | 0.058 |
| Satisfaction with living situation (Ref. neither satisfied nor dissatisfied) |  |  |  |  |  |  |
| Satisfied | **2.62** | **2.14; 3.10** | **< 0.001** | **0.51** | **0.12; 0.91** | **0.011** |
| Dissatisfied | **−1.55** | **−2.25; −0.85** | **< 0.001** | **−0.66** | **−1.23; −0.10** | **0.021** |
| Discrimination due to origin (Ref. rarely) |  |  |  |  |  |  |
| Never | **1.91** | **1.45; 2.38** | **< 0.001** | **0.68** | **0.08; 12.8** | **0.026** |
| Often | **−1.08** | **−2.03; −0.13** | **0.026** | −0.41 | −0.88; 0.07 | 0.092 |
| Feeling of being welcome (Ref. in some respects) |  |  |  |  |  |  |
| Completely or predominantly | **2.45** | **1.75; 3.15** | **< 0.001** | 0.44 | −0.16; 1.05 | 0.152 |
| Barely or not at all | **−2.19** | **−3.65; −0.72** | **0.003** | −0.00 | −1.22; 1.21 | 0.997 |
| Feeling of missing people from the country of origin (Ref. sometimes) |  |  |  |  |  |  |
| Rarely or never | 0.20 | −0.51; 0.91 | 0.584 | **0.68** | **0.08; 1.28** | **0.026** |
| (Very) often | −1.10 | −1.63; −0.56 | < 0.001 | −0.41 | −0.88; 0.07 | 0.092 |
| Worries about not being able to stay in Germany (Ref. some worries) |  |  |  |  |  |  |
| Great worries | −0.91 | −1.48; −0.34 | 0.002 | −0.39 | −0.85; 0.07 | 0.099 |
| No worries | 1.84 | 1.32; 2.36 | < 0.001 | −0.00 | −0.44; 0.43 | 0.987 |
| Worries about not being able to return to country of origin (Ref. some worries) |  |  |  |  |  |  |
| Great worries | −1.14 | −1.82; −0.47 | 0.001 | 0.04 | −0.51; 0.59 | 0.887 |
| No worries | 1.31 | 0.81; 1.81 | < 0.001 | **0.85** | **0.45; 1.25** | **< 0.001** |
| Survey year (Ref. 2016) |  |  |  |  |  |  |
| 2017 | 0.62 | −0.07; 1.30 | 0.078 | 0.27 | −0.29; 0.82 | 0.345 |
| 2018 | **1.47** | **0.88; 2.07** | **< 0.001** | −0.20 | −0.69; 0.30 | 0.437 |
| 2019 | **1.96** | **0.62; 3.30** | **0.04** | −1.09 | −2.28; 0.10 | 0.074 |
| Initial sample | **🗸** | | | **🗸** | | |
| Constant | **46.37** | **44.85; 47.90** | **< 0.001** | **64.43** | **63.15; 65.72** | **< 0.001** |

CI: confidence interval

^a^ Without Syria, Iraq and Afghanistan

^b^ Another/no residence title not shown

^c^ Permission to stay pursuant to Section 55 of the German Asylum Law (asylum seekers) or temporary suspension of deportation according to section 60a of the German Residence Act

^d^ Settlement permit according to Section 26 sub-section 3 of the German Residence Act

^e^ Residence permit according to Section 25 sub-section 1 of the German Residence Act (persons entitled to asylum), according to Section 25 sub-section 2 of the German Residence Act (persons with refugee status), according to Section 22 or Section 23 of the German Residence Act (admission on humanitarian grounds), or residence permit pursuant to § 23a or § 25 sub-section 3, 4 or 5 of the German Residence Act (admission on other humanitarian grounds)
